# Post kala-azar dermal leishmaniasis: A threat to elimination program

**DOI:** 10.1371/journal.pntd.0008221

**Published:** 2020-07-02

**Authors:** Mallikarjuna Rao Gedda, Bhawana Singh, Dhiraj Kumar, Abhishek Kumar Singh, Prasoon Madhukar, Shreya Upadhyay, Om Prakash Singh, Shyam Sundar

**Affiliations:** 1 Infectious Disease Research Laboratory, Department of Medicine, Institute of Medical Sciences, Banaras Hindu University, Varanasi, India; 2 Center for Cellular Engineering, NIH Clinical Center, Bethesda, Maryland, United States of America; 3 Department of Zoology, Rameshwar College, BRA Bihar University, Muzaffarpur, India; 4 Department of Biochemistry, Institute of Science, Banaras Hindu University, Varanasi, India; Centro de Pesquisa Gonçalo Moniz-FIOCRUZ/BA, BRAZIL

## Abstract

Leishmaniasis remains a public health concern around the world that primarily affects poor folks of the developing world spanning across 98 countries with mortality of 0.2 million to 0.4 million annually. Post kala-azar dermal leishmaniasis (PKDL) is the late skin manifestation of visceral leishmaniasis (VL). It has been reported that about 2.5% to 20% of patients recovered from VL develop PKDL having stilted macular or nodular lesions with parasites. In the Indian subcontinent (ISC), it manifests a few months after recovery from VL, though in Africa it can occur simultaneously with VL or a little later. New cases of PKDL are also observed without prior VL in the ISC. These individuals with PKDL represent an important but largely neglected reservoir of infection that perpetuates anthroponotic *Leishmania donovani* transmission in the ISC and can jeopardize the VL elimination program as these cases can infect the sand flies and spread the endemic. Therefore, it becomes imperative to eradicate PKDL as a part of the VL elimination program. With the limited treatment options besides little knowledge on PKDL, this review stands out in focusing on different aspects that should be dealt for sustained VL elimination.

## Background

Leishmaniasis or kala-azar, a protozoan parasitic human disease caused by *Leishmania* parasite in the tropical and subtropical regions through the bite of sand fly (*Phlebotomus* spp). Out of 54 known species of this parasite, only 21 species are known to cause the disease in 98 countries, and around 350 million subjects are at the risk of infection [1]. Based on its clinical manifestations, this disease occurs into self-healing cutaneous leishmaniasis (CL), skin mucosal ulcers forming mucocutaneous leishmaniasis (MCL), and fatal visceral leishmaniasis (VL), which may be followed by a dermal sequel called PKDL. PKDL, which can be confused with leprosy [2], develops after six months and sometimes up to 5 years following the previous VL incidence, and 15% of PKDL cases have shown no prior kala-azar infection [3]. But a hospital-based retrospective study on Indian PKDL subjects showed that 20% of cases had no antecedent of VL [4]. The diagnosis of PKDL can be established through slit-skin smears (SSS), culture, and/or polymerase chain reaction (PCR) [5]. The clinical manifestation of VL and PKDL differs vastly, where the former include prolonged fever, hepatosplenomegaly, anemia, and weight loss, and the latter with macular, papular, or nodular lesions [6]. The geographical distribution of PKDL involves the East Africa zone (Sudan) having papular or nodular lesions and the South Asia zone (India, Bangladesh and Nepal) with widespread polymorphic lesions (co-occurrence of macules and patches besides papulonodules) [7, 8] having numerous intracellular and extracellular *Leishmania donovani* bodies (LDBs) [9].

## PKDL: A hidden agenda of the *L*. *donovani* parasite

This stigmatizing vector-borne disease often remains untreated due to patients’ reluctance or non-compliance with the therapy. The PKDL hypomelanotic lesions, especially the papulo-nodules with centripetal distribution [9], which are rich in the parasite load, may also play a vital role in the transmission of VL. The knowledge of previous studies gave five hypotheses for the development of PKDL, i.e., through reinfection or relapse, which includes the pentavalent antimonial drugs, genetic susceptibility of the host, UV-induced skin damage, organ-specific failure of memory T cell and reinfection or persistence of the parasite [6].

### Drugs towards the development of PKDL

Previous epidemiological and clinical data have suggested that there may be a link between sodium antimony gluconate (SAG) and subsequent development of PKDL [10]. The concept has been deemed to be feasible in the case of Sudan, Bangladesh, and Nepal, with 100% patients of PKDL received SAG therapy [11–13]. While in India, 73% of patients developed PKDL with SAG treatment on nine years follow-up of VL [14], and the remaining 27% has been contributed by other antileishmanial drugs, i.e., amphotericin B (AmB) or its liposomal formulation, miltefosine, and paromomycin. One of the exciting things is that SAG is still used for the treatment of PKDL, with a long (120 days) and arduous regimen. However, due to the rise in antimony resistance in India, miltefosine, and AmB have been extensively used for the treatment of VL subjects [15]. In a clinical study, when AmB administrated at 20 and 15 mg per kg for the treatment of VL, the former concentration has effectively minimized the PKDL development while the latter drug dose administration progressed into PKDL [16]. Alongside, several studies have shown that VL patients receiving paromomycin, miltefosine, and combination therapy of miltefosine and AmB developed PKDL infrequently [14, 17, 18].

Additionally, in vitro studies to test the minimum antileishmanial activity of five classical leishmanial drugs on clinical isolates of PKDL from India have revealed the lowest susceptibility to SAG against the parasite [19]. Although the data may be incriminating SAG directly or indirectly for PKDL development, further studies may give some final pieces of evidence. There are pieces of evidence for potency of the lower dose of the drug for a shorter period could eliminate the visceral parasite without affecting the parasite load in the dermis, which can be 100% cleared at a higher dose. Hence, PKDL cure could be related to SAG along with its dosage of use, which can be explained by further pharmacokinetics and pharmacodynamics studies.

Also, the treatment with SAG has shown sustained levels of transforming growth factor beta (TGF-β), interleukin (IL)-10, heme oxygenase 1, and glutathione in comparison with AmB or miltefosine, which may eventually help in the parasite persistence inside the host [20–22]. The dysfunctional peroxisome inside the host due to the SAG treatment could also be a contributory factor for PKDL incidence [23]. Additionally, SAG induced genetic alterations may have enhanced degree of fitness for the parasite [24] through enhanced expression levels of promastigote surface antigen (PSA2), glycoprotein 63 (gp63) and alleviated amastigote antigen 2 (A2) expression [25, 26], which may eventually show enhanced dermatotropism for incidence of PKDL. A retrospective cohort study postulated that environmental factors like long term exposure of arsenic in the groundwater could also act as an additional factor for the development of PKDL in VL patients treated with SAG or other drugs treated VL patients [27]. Hence, a holistic knowledge of every aspect of PKDL will help us in combating this disease.

### Genetic susceptibility of the host towards PKDL

The genetic analysis of VL in murine models and human subjects has been thoroughly characterized to date, but this is not the case for PKDL [28–31]. According to a genetic study of PKDL patients in Sudan, one question remains unanswered as to why some of these VL patients grow PKDL while others do not. Some studies have also reported that interferon gamma (IFNγ) receptor polymorphism has a significant association with the development of PKDL [32, 33]. IFNγ receptor polymorphism leads to decreased responsiveness to the IFNγ, although it is present in higher levels. The equilibrium between proinflammatory and antiinflammatory cytokines is very crucial for pathogenesis during *Leishmania* infection [34], although no correlation has been found between promoter polymorphism of IFNγ or IL-10 and disease susceptibility [33, 35].

Moreover, this IFNγ receptor polymorphism or decreased expression levels of IFNγ receptor [36] may lead to parasite survival and growth due to failure to respond to enhanced IFNγ levels. A study in Sudan, using 30 PKDL patients biopsies, have shown uniformly alleviated expression of IFNγ and its receptor in dermal tissue, which could explain parasite’s persistence and is consistent with the previous demonstration of genetic association with IFNγ receptor polymorphism [37]. Beside the IFNγ receptor, higher levels of plasma C-reactive protein (CRP) has been observed in Sudan PKDL patients [38], which remains to be solved whether it’s a polymorphism in the gene or its promoter that promotes the development of PKDL. Besides PKDL, gene-like Ficolin-2, solute carrier family 11a and mannose-binding lectin 2 polymorphisms at the promoter regions and enhanced expression of mannose-binding lectin 2 were stated to increase the susceptibility to CL and VL, respectively [39–43]. Additionally, rigorous studies on PKDL emphasizes for epigenetic modification(s) in the above-discussed genes, may help in further research of PKDL susceptibility.

Studies based in India by Dey and colleagues and Gannavaram and colleagues on the *L*. *donovani* isolate from PKDL and VL patients, have identified polymorphisms in a well-defined genetic locus (LdP13 & β-tubulin) among the parasites causing the visceral and dermal manifestations [44, 45]. These results have been verified using molecular techniques such as random amplified polymorphic DNA (RAPD)-PCR and PCR- single-strand conformation polymorphism (SSCP) to investigate the single base differences for the detection of polymorphisms in *Leishmania* species and strains [46, 47]. Additionally, the comparative gene expression profiling of genes (such as DEAD-box RNA helicase, iron superoxide dismutase b, putative phosphodiesterase, hypothetical protein from the isolates of both VL and PKDL and cell surface proteins up-regulation in PKDL) demonstrates that further studies are needed to know the critical insight of the parasite differences for the disease manifestations by the parasite [25, 46, 47]. However, genetic polymorphisms in Sudan *L*. *donovani* isolates from VL and PKDL using amplified fragment length polymorphism (AFLP) and kDNA minicircle sequencing haven’t reported anything significant [48]. More and more studies are needed to crack the genetic variations between the clinical isolates of VL and PKDL in Sudanese population beside genetic differences between the clinical isolates from the Indian subcontinent and Sudan.

### UV-induced development of PKDL

The typical PKDL lesions appear on the face, ears, and arms, which are regularly exposed to the sunlight leaving back the unexposed areas such as the scalp and chest without any lesions. This has also been in support of the theory that the UV light plays one of the crucial roles in the PKDL susceptibility [49], which highlights the presence of photosensitivity in subjects with PKDL. UV light acts as a potent immunosuppressive agent by damaging the antigen-presenting epidermal Langerhans cells (E-LC), which finally affects the alloantigen and hypersensitivity responses [50]. This immunosuppression by UV light, especially UVB (280–320 nm) induces keratinization mainly via vitamin D3 modulation or through *trans*- to *cis*-urocanic acid photoisomerization [51, 52]. Furthermore, *cis*-urocanic acid exerts its effect on keratinocytes and lymphocytes by modulating their proinflammatory and anti-inflammatory cytokines, especially IL-10, which is immunosuppressive.

About 40% of the adolescent population is sensitive to UVB, which may also account for the PKDL susceptibility in this population with cured VL [49]. This photosensitization affects all the cells types of the dermis but mainly affecting E-LCs leading to its dysfunction through alteration in morphology and their characteristic dendritic pattern with reduced major histocompatibility complex (MHC) class II and costimulatory molecules (CD80 and CD86), which eventually affects the antigen-presenting property [53–56]. Studies on Indian PKDL patients have showed that the pattern of alleviation in expression of c-stimulatory molecules [21, 57] has been in parallel with the previous results from the UV irradiation [51] with enhanced levels of tumor necrosis factor alpha (TNF-α) and IL-10 cytokines in the lesions, which supports UV role both in disease pathogenesis and immunosuppression [36, 58]. Additionally, IL-10 has been attributed to the pathogenesis of VL by rendering the macrophages inactive for activation signals for their antileishmanial activity, and this has been in parallel in case of PKDL from the results with proparasitic milieu with increased lesional IL-10 [59].

Vitamin D3’s role in the pathogenesis of PKDL seems a bit controversial because it can act as an immunomodulator of macrophages; on the other hand, it also inhibits toll-like receptor (TLR)based activation of macrophages [6]. But a recent clinical study on Indian PKDL patients has shown that vitamin D–regulated cationic antimicrobial peptide; cathelicidin-decreased expression levels in untreated PKDL subjects [60]. On AmB treatment, the PKDL macrophages showed VDR-dependent cathelicidin positive induction through TLR2 and IL-1β but not TLR4, leading to antileishmanial effect and macrophage activating potential.

### PKDL susceptibility through an organ-specific failure of memory T cell?

The reinfection of the *Leishmania* parasite has been sharply denied by lifelong and sharp memory responses in humans [61]. However, under conditions of immunosuppression, this immunity induced by the parasite gets impaired, leading to relapse of the *Leishmania*-induced disorders [62]. The qualitative and quantitative aspects of the parasite in the liver and spleen, where the *Leishmania* resides, influence the host immunity [63]. These are challenged by the PKDL infection (acquired from VL) despite systemic protective immunity acquirement known through the cytokine levels and T-cell responses in whole blood or peripheral blood mononuclear cells [64, 65], and organ-specific failure of the memory cell in the skin leads to susceptibility. This may be due to the presence and persistence of T helper (Th)2 response in the skin biopsies with systemic Th1 response due to the VL treatment [66].

The disease outcome in PKDL patients of South Asia and Sudan differ significantly in the result of the disease with no resolution without the treatment, while in Africa lesions regress spontaneously in the majority of patients [11]. However, Sudanese PKDL subjects showed a shorter lag period, which may be due to their active immune memory responses that might mimic post-VL, whereas, in South Asia, PKDL subjects show an extended lag period, which may be due to their immune anergy in eliciting an antigen-specific immunological response [65]. It has also been postulated that the self-healing ability of Sudanese PKDL is due to the presence of augmented levels of effector memory T cells in the skin, which has been found alleviated in the case of the non–self-healing nature of Indian PKDL [61, 67, 68]. This hypothesis may become feasible if comparative studies on memory responses have to be done between self-healing Sudanese PKDL and non–self-resolving Indian PKDL. In Indian PKDL, the *Leishmania* antigen or phytohaemagglutinin (PHA) response in the circulating CD4+ T cells levels were intact but impaired functionally in case of CD8+ T cells [65]. Another study on the Indian subjects showed that the presence of *Leishmania* antigen results in elevated levels of both activated CD4 and CD8 T cells with predominant granzyme B secretion, which showed a critical role of cytotoxic cells in resistance to *L*. *donovani* infection in polymorphic PKDL [69].

Moreover, the histopathological studies on IL-2 receptor (i.e., CD25 on regulatory T [Treg] cells have been found reduced in the skin biopsies of Sudanese PKDL while increased in the case of Indian variants [58, 70]) might play an essential role in pathogenesis. Furthermore, cutaneous lymphocyte antigen (CLA), which is required for self-resolving CL, could also be correlated with nonhealing outcomes of Indian PKDL [71, 72]. Notably, the granuloma formation is shallow in case of Indian PKDL with a decreased proportion of CD4+ T cells [6, 58, 64, 67, 73], which may cause impaired tissue-specific immunity leading to nonhealing characteristic. A study using cDNA microarray and quantitative polymerase chain reaction (qPCR) studies of PKDL biopsies have shown a remarkable effect on host cellular gene expression with enhanced Th17 response [74]. IL-17 stimulation increased the production of TNF-α and nitric oxide (NO) by peripheral blood mononuclear cells (PBMCs) from PKDL patients may point to a direct role of T regulatory cells in parasite persistence [75].

### PKDL susceptibility through reinfection or persistence of the parasite?

Many infectious diseases are characterized by persistence or reinfection of the pathogen after medical cure such as in the case of tuberculosis, protozoan diseases, and viral infections [76]. In endemic areas, leishmaniasis has shown recurrence due to parasite persistence after the curing of clinical subjects [77, 78]. Hence, an approach that would characterize and compare the PKDL parasite with the parental type by using genomics and proteomics, which would establish whether it has been through parasite persistence or reinfection. A previous animal study on mice model has shown that these parasites were similar to the parental type [77] favors persistence, which couldn’t be translated in case of humans due to genetic heterogeneity between strains isolated from VL and PKDL in the district of Bihar state, India, pointing towards reinfection [45, 76, 78]. However, the majority of PKDL occurring in patients with past VL favors reactivation of previous infection. A de novo whole-genome sequence study and their annotation of a PKDL *Leishmania* strain in Indian patients have postulated a possibility of endosymbiotic infection and superinfection for PKDL manifestations [79]. In Sudan, the PKDL pathogenesis may be due to the persistence of the parasite as the majority of VL patients develop PKDL during or immediately after the episode of VL [6].

## Biomarkers for PKDL

Biomarkers are a wide range of diagnostic indicators that indicate the body's medical condition and may include physical signs seen on clinical inspection, necessary chemical tests, and more detailed blood and other tissue monitoring [80]. The inadequacy of the studies on PKDL hampers the identification of biomarkers of PKDL. Biomarkers can be parasitological, serological, immunological, and pathological or from repeated clinical assessments [81]. Besides this, Table 1 summarizes the currently available biomarkers used for the diagnosis of PKDL. Additionally, the table also specifies some of the characteristics ranging from their respective detection techniques and the region of the study to the biomarker evaluation score. Laboratory tests provide qualitative testing methods but still have drawbacks, although qPCR tends to be the preferred molecular tool for assessing parasite load in drug studies. Results from various studies are considerably heterogeneous, including measurements in clinical types (macular and papulo-nodular); this may be due to regional differences, targeted genes, clinical characteristics (duration, size, self-healing), and sampling techniques, among others. qPCR in a SSS or aspirate can be used to monitor the parasite load during treatment and is a most preferred biomarker to measure the response to treatment objectively and is more patient-friendly than a tissue biopsy.

**Table 1:** Summary of the currently available biomarkers used for the diagnosis of PKDL.

Marker category	Biomarker	Detection technique(s)	Matrix(ces)	Region(s)	Clinical presentation(s) of leishmaniasis	Time until normalcy	Biomarker Evaluation Score				
							Specificity	Sensitivity	Additional sensitivity	Geographical applicability	References
Direct markers for parasite detection	Parasites in lesion biopsy specimen	qRT-PCR, NASBA	Lesion biopsy specimen	Netherlands/India/Germany/ Israel/Brazil	CL/PKDL	+	++	++	?	++	[76, 82–90]
Indirect markers for Cytokines	IL-10	CBA/ELISA/multiplex biometric immunoassay	Serum	India/Brazil/Sicily/Ethiopia	VL/PKDL	+	-	++	+	++	[36, 82, 91–101]
	IFN-gamma	CBA/ELISA/multiplex biometric immunoassay	Serum	India/Brazil/Sicily/ Ethiopia/Sudan	VL/PKDL	+	+	++	?	++	[36, 92–95, 97, 98, 101–106]
	TGF-β	RT-PCR/immunohistology	Biopsy specimen	India/Brazil/Mexico	CL/PKDL	?	?	+	?	++	[36, 107]
	TNF-α	CBA/ELISA/ immunoradiometric assay kit	Serum	Brazil/India/Ethiopia/Sudan	VL/PKDL	++	++	+	?	-	[36, 98, 102, 104–106, 108–113]
Cell surface molecules and circulating receptors	sIL-2R	ELISA	Serum	Brazil/Sicily	VL/PKDL	-	?	++	+	++	[108, 114–117]
Other proteins	Arginase	Colorimetric assay	PBMCs/lesion biopsy specimen/ serum	Brazil/India/Ethiopia	VL/CL/PKDL	++	?	+	-	?	[21, 118–121]
	CTLA-4 (CD152)	RT-PCR	Lesion biopsy specimen	India	PKDL	-	?	+	?	?	[122]
	Foxp3	RT-PCR	Lesion biopsy specimen	India	PKDL	+	?	+	+	?	[122, 123]

CBA, cytometric bead array; CD, cluster of differentiation; CL, cutaneous leishmaniasis; CTLA-4, cytotoxic T-lymphocyte-associated protein 4; ELISA, enzyme-linked immunosorbent assay; Foxp3, forkhead box protein P3; IL, interleukin; IFN, interferon; NASBA, nucleic acid sequence-based amplification; PKDL, post kala-azar dermal leishmaniasis; RT-PCR, real-time polymerase chain reaction; VL, visceral leishmaniasis; TGF, transforming growth factor TNF, tumor necrosis factor.

## Diagnostic and detection approaches of PKDL

The successful detection of infective agents in PKDL primarily relies on the sensitivity, specificity, ease of use of the method, and its applicability in the field. Presently, the diagnosis of PKDL has restricted principally to referral hospitals and a research centers with adequate laboratory settings. Even today, primary diagnosis of PKDL mainly relies on clinical assessments, including the history of VL, typical skin lesions, evidence of past VL by antileishmanial antibody, and exclusion of skin fungal disorders and leprosy. At present, the following methods are in practice for the accurate detection of PKDL.

### Histopathology

Diagnosis of PKDL through histopathology uses hematoxylin and eosin (H&E) staining for the demonstration of LDBs in skin specimens and is considered as a gold standard. However, the degree of positivity for LDBs varies from 67% to 100% in nodular lesions, 36% to 69% in papular lesions and 7% to 33% in macular lesions [124, 125]. Furthermore, histopathological observations elucidated varying proportions of lymphocytes, macrophages, and plasma cells in different clinical forms of PKDL [126]. The epidermal layer of macular PKDL is healthy and shows an insufficient number of LDBs, resulting in a poor demonstration. Nodular PKDL shows atrophied epidermis with abundant LDBs and can be observed easily in such lesions. Recently, *Leishmania*-specific monoclonal antibodies have been used in immunohistochemistry (IHC) assay, and it increased the LDBs localization (80%) as compared to H&E staining (50%).

The morbidity of PKDL in the Indian subcontinent is prolonged irrespective of the morphology of lesions. Several histopathology studies have gone through the infected skin lesion tissues having numerous intracellular and extracellular *L*. *donovani* bodies [5, 9, 58, 73, 127–129]. A retrospective study, with biopsies of 88 skin and 16 mucosal lesions of PKDL patients were studied from 2004 to 2011 in Bihar has shown histo-morphological patterns having follicular plugging, grenz zone, and dermal infiltrates arranged in three modes: diffused infiltrates (46.6%), perivascular and perifollicular infiltrates (27.3%), and superficial perivascular infiltrates (18.1%) [127]. A light microscopical, immunohistochemical, and ultrastructural study on skin lesions showed dermal infiltration by lymphocytes and macrophages and small plasma cells, with a majority of CD3 T-cells having the more significant part of CD4 T-cells over CD8 T-cells [58]. Additionally, it has also been shown that human leukocyte antigen– DR isotype (HLA-DR), intracellular adhesion molecule (ICAM)-1, and parasite antigens are expressed by degenerating basal keratinocytes that closely interacted with the CD4 T-cells. Another retrospective study from India, based on clinicopathological and immunological changes have shown that cellular infiltration in skin lesions are mononuclear cells chiefly histiocytes with vacuolation, minimal plasma cells, and many lymphocytes [128]. Although these lesions disappeared on treatment with SAG, their microscopic studies have shown persisted mononuclear cells.

### rK39

The rK39, a rapid dipstick test, is a promising method for the diagnosis of VL as well as PKDL, with the only limitation differentiating between past and present infection. K39 is a conserved epitope of amastigote form of *L infantum*. In the Indian subcontinent, circulating anti-K39, immunoglobulin G (IgG) can be detected in 95% to 100% of patients who have had kala-azar. Using this method, fingerstick-obtained blood and serum samples tested from Indian subjects demonstrated positive in 362 patients with aspirate proven kala-azar in a study from Nepal.

### PCR

Amongst the molecular methods, PCR has been categorized as the most promising technique for the clinical diagnosis of PKDL. The detection of *Leishmania* DNA is possible in various clinical samples, and maximum sensitivity can be achieved by multicopy PCR amplicon. Several genes, like ribosomal RNA, kinetoplast DNA (kDNA), mini-exon-derived RNA (med RNA), β-tubulin, and gp63, have been extensively targeted for PCR of PKDL diagnosis [130]. PCR-based diagnostic approaches for PKDL are adapted from VL diagnosis because of the limited number of reports employing PKDL [131]. Furthermore, PCR-based methods can detect *Leishmania* parasites in patients with low levels of parasitemia and even a few weeks before the appearance of clinical symptoms. Specimens like skin biopsy of lesions, peripheral blood (mostly buffy coat), slit aspirates, or lymph node aspirates are used for PKDL diagnosis.

In Indian patients, a species-specific PCR assay based on kDNA was developed for VL and PKDL. The method provided a detection limit of 1.0 fg of purified *L*. *donovani* DNA (equivalent to less than one parasite) or 10 fg of parasite DNA (equivalent to one parasite) in existence of 10 million-fold excess of human DNA [132]. Also, the same study reported the presence of the parasite in 45 of 48 PKDL skin biopsies with a sensitivity of 93.8%. Another research article from Sudan employed PCR and reported a sensitivity of 82% in slit aspirate and 83% in lymph node aspirate, respectively [131]. Furthermore, a less invasive nested PCR method was developed for the diagnosis of PKDL. The nested PCR effectively detected parasite DNA in slit aspirates of 27 out of 29 cases with much higher sensitivity as compared to primary PCR [133, 134]. Isolated histiocytes infiltrate from CL patient’s dermal lesions using laser capture microdissection and amplified the kDNA for the presence of the parasite in extracted DNA. Interestingly, the lesions showed no evidence of *Leishmania* on giemsa staining but were positive by PCR for the *Leishmania* parasite. Thus, microdissection of infected dermal cells from PKDL and subsequent analysis can improve the diagnosis PKDL. Proper precautions like the risk of contamination, false-positive results, and positive and negative control must be included for avoiding improper diagnosis.

### Direct agglutination test

**Direct agglutination test** (DAT) is a simple and reliable technique, which has high sensitivity and specificity, making it suitable for both field and laboratory use. The test can be carried out by using whole blood, plasma, or serum. This method uses trypsin-treated and Coomassie Brilliant Blue–stained whole promastigotes either as a suspension or in a freeze-dried form. The freeze-dried form of DAT antigen is heat stable, which facilitates its use in the field. The main disadvantage of the DAT test is the longer incubation time of approximately 18 hours and the requirement for serial dilution of blood samples. Also, DAT has no prognostic measure for evaluating the parasitological cure of the disease as the positive test results may persist for several years after the treatment. The effectiveness of DAT in the diagnosis of PKDL was first demonstrated by El Harith and colleagues [135] using antigen prepared from indigenous isolates. By using indigenous isolates, the DAT was found capable of distinguishing CL, MCL, and other skin disease conditions like leprosy, vitiligo, psoriasis, and cutaneous tuberculosis. DAT has been used in the diagnosis of VL in India with high sensitivity (96% to 98%) and specificity (100%) [136] [137]. With reference to its use for PKDL diagnosis, DAT has not been used very widely.

One of the studies has found DAT to be 100% sensitive and specific for the diagnosis of PKDL in Nepal [138]. In another study on the diagnosis of VL in Nepal, DAT was found to be 91% sensitive and 69% specific, with a cut-off titer of 1 to 6,400 in a hospital-based study [139]. However, in a field-based study using the same cut-off titer of 1 to 6,400, DAT was 100% sensitive and 93% specific [140]; and, in Sudan, sensitivity and specificity were found to be 95.9% and 99.4%, respectively, at a cut-off titer of 1 to 8,000 [141]. However, as all of those patients were PKDL, in whom the parasite remained for the extended period, this increased the sensitivity and specificity of the DAT to 100%.

### Real-time PCR

Real-time PCR as a tool for biomarkers discovery in VL was initially performed and described by Vallur and colleagues[142]. Quantitative real-time PCR allows the continuous monitoring of the PCR product accumulation during the amplification reaction. The estimate of the relative load of parasites in the various samples can be obtained from the quantification of the template DNA. The real-time PCR assay is a reliable and noninvasive tool for the diagnosis of VL and PKDL patients [142]. The diagnosis of PKDL may be done by the presence of leishmanial parasites by microscopy in a micro-biopsy, SSS, or fine needle aspirate (FNA). Parasites can be found more readily in mixed papulonodular lesions (up to 90%) than in the macular lesions (up to 40%); biopsies from the buccal mucosa and the tongue have higher yield [128, 143]. In a recent study comparing the tissue biopsy and SSS, all PKDL cases with macular lesions (*n* = 4) were negative in microscopy, whereas in papular lesions, 2 of 17 and 10 of 20 were positive in microscopy, in SSS and biopsy, respectively; for the nodular lesions, 13 of 26 and 20 of 26 cases were microscopically positive in SSS and biopsy, respectively [144].

qPCR is more sensitive than microscopy, but it requires a well-equipped laboratory. Real-time PCR or qPCR allows detection and quantification of the parasite number [145]. Mary and colleagues [146] developed a real-time PCR assay to quantify *Leishmania’s* kDNA and optimized the sensitivity to detect up to 0.0125 parasite per ml in the blood. There are some studies on the qPCR as a tool to detect parasites after treatment. Patients having higher parasite load as quantified by the qPCR are at a higher risk of relapse; in a study comprising 30 patients, one month after the treatment, 26 of 30 were negative in qPCR, while four showed leftover parasites, of whom two relapsed [143]. In a study of 15 miltefosine treated patient samples, after 60 or 90 days posttreatment, all 15 became parasitologically negative as quantified by the qPCR from an SSS [147]. In another study, 17 of 19 patients became negative by qPCR one month after treatment with SSG or miltefosine; in two patients, a leftover parasite load was found (7 and 8 parasites per μl slit aspirate, respectively); one of these patients relapsed [148]. It is evident that the real-time PCR assay is useful for epidemiological and diagnostic purposes and to assess the parasite burden in symptomatic as well as asymptomatic carriers.

### Xenodiagnosis

The only recognized proof for reservoir infectiousness is xenodiagnosis, which consists of feeding lab-reared sandflies on the presumptive reservoir, resulting in the infection in the fly [149]. Several xenodiagnosis studies performed over two to three decades lead to the finding that PKDL patients constitute a crucial interepidemic reservoir of *Leishmania* [150, 151]. In the scope of local elimination of VL, Mondal and colleagues presented quantitative data on the significance of PKDL patients as potential reservoirs by reporting the results of xenodiagnosis from 47 PKDL and 15 VL patients. The study depicts that, prior to PKDL onset, all the patients have been treated for VL, 81% with pentavalent antimonial drugs and out of 47, 26 (55.3%) had macular, papular, or maculopapular lesions, while 21 (44.7%) had nodules or a combination of nodules and macules as confirmed by skin biopsy qPCR and microscopy. The factors associated with positive xenodiagnosis includes nodular lesions, younger age, positive microscopy, and skin parasite load. They showed that nodular PKDL was more likely (86%) and macular PKDL less likely (35%), resulted in infected sandflies as compared with VL (67%), and concluded that PKDL is nearly equally infectious as that of VL. This conclusion was consistent with the fact that PKDL patients go untreated for years due to scarcity of adequate diagnostic tools and resemblance with leprosy [2], accounting for their higher cumulative transmission potential than that of VL patients. Although the VL elimination program is on the verge of its accomplishment (1 case per 10000 persons per year), PKDL stands as a major obstacle as the contribution of PKDL cases nearly triples after intensive WHO interventions highlighting the need for prompt PKDL control program [152, 153].

## Classical therapeutics of PKDL and its limitation

Although the PKDL lesions heal spontaneously in the majority of cases (in Sudan), however, in the Indian subcontinent, it manifests in 2% to 20% of VL subjects after six months to several years after treatment [154]. Until recently, SSG (20 mg/kg per day for 20 days per month for 6 months) was the treatment of choice for PKDL. However, long hospitalization periods, toxicity (cardiac toxicities, arthralgia, etc.), and daily painful injections have limited its use [155–157]. These limitations lead to inadequate medical care and patient compliance.

The earliest evidence for the cure of PKDL cases with conventional AmB dates back to 1974 when Yesudian and colleagues used 717.5 mg for three months and yielded 50% cure rates [158]. Later in 1997, Thakur and colleagues used 60 to 80 infusions of 1 mg/kg (3 to 4 cycles of 20 infusions at an interval of 20 days between two cycles), which cured all the cases and was found to be superior to SSG with high cure rates, but high cost, as well as toxicity issues, and prolonged hospitalization, limited its use as a treatment option for PKDL [159]. AmB was preferred due to less toxicity as compared to SSG. However, the variable success rates of AmB first tested for curing PKDL (25 mg, alternating days for the varying duration) in two cases. Later, AmB (10 mg/kg for 20 days) reported for 64% relapsed cases. These issues led to patient noncompliance, and such matter can serve as potential reservoirs for drug-resistant parasites, therefore hampering the disease management strategies. Recently, 20 infusions for a total of three courses of AmB (0.5 mg/kg) have yielded reasonable cure rates; however, nephrotoxicity limited its acceptability [160].

Liposomal formulation of AmB (AmBisome) was first used for a small case study in Sudan for treating two patients with PKDL [161]. Later, it was used in six infusions of 5 mg/kg dose, for three weeks, curing 96% of macular cases of PKDL. Upon a follow-up after 12 months, the macular and papular lesions healed entirely with significant skin repigmentation. AmBisome (2.5 mg/kg per day for 20 days) has shown safety and efficacy in the treatment of SSG resistant cases with 83% cure rates [162]. However, the potential threat to the emergence of hypokalaemia induced rhabdomyolysis raised concerns regarding its usage [163]. Another prospective cohort study in Bangladesh, with a short-course (15 mg/kg) AmBisome regimen was efficacious and improved lesions in 89% of cases with no serious adverse events [164]. A similar study from Sudan (2.5 mg/kg dose for 20 days) brought regression in the papular and macular lesions, with no adverse events [162]. However, a cohort study from the Bihar state of India has reported for the shorter median time for the development of PKDL in cases that were previously treated with 20 mg/kg AmBisome and, therefore, called upon for proper follow-up of treated cases for the imminent risk of PKDL development [165].

In 2006, miltefosine was used for treating PKDL, which took a more extended treatment duration than the VL treatment due to its limited skin permeability. It has been used as the first-line regimen as per WHO recommendation, with a total period of 12 weeks for treating PKDL in the Indian subcontinent [166]. This regimen was tested in a multicentric trial with the 61% to 88% (12 weeks) and 53% to 90% (8 weeks) cure rates [167]. However, later in 2015, 16 weeks of treatment was proven to be more productive than 12 weeks of therapy since the latter possessed the risk for the emergence of relapse cases [168]. Miltefosine has been known to trigger heightened proinflammatory Th1 responses in the Indian cohort, thus superseding the SSG based regimen for PKDL treatment [169]. However, the efficacy has recently been declining due to patient noncompliance, which is associated with frequent gastrointestinal complications in cases [170].

Presently, PKDL patients in the Indian subcontinent are treated with 50 mg miltefosine (for 12 weeks, twice a day); longer treatment duration (up to 16 weeks) led to improved cure rates of 96% to 100%, but many gastrointestinal side effects posed the hurdle [171]. Hence, it would be too early to conclude that prolonged treatment regimens are safe. In light of the evidence from pharmacovigilance studies, miltefosine usage has shown severe adverse reactions [170]. Furthermore, increasing incidence of miltefosine resistance has been seen even in PKDL cases [172]. The protracted treatment regimens invariably led to either nonacceptance or poor patient compliance.

After the approval of paromomycin for curing VL, it emerged as another low-cost alternative for the treatment of PKDL. A cohort study in the endemic area of Bihar (India) reported for low cure rates with 45 intramuscular injections of paromomycin, without any significant side effects [173]. Further, the emergence of paromomycin resistance has also been reported by Singh et al. in 2012 [174]. However, the drug was used in Sudan, in combination with SSG, and yielded promising cure rates as compared to SSG monotherapy (97% vs 90%) with shorter treatment duration (34 days versus 42 days). Thus, combination therapy emerged as a low-cost therapeutic option for PKDL treatment [175]. Combination therapy using AmB with miltefosine reduced the treatment duration and dosage with better tolerance as well as compliance rates [176]. Likewise, in contrast to 12 weeks of monotherapy with miltefosine, its combination with paromomycin had shorter treatment duration (60 days), decreased the hospitalization period, and was cost-effective [177]. Combination therapy using SSG with rifampicin showed excellent cure rates, patient compliance and minor side effects [178]. However, a combination of two antifungal compounds, terbinafine with itraconazole, failed to cure PKDL in Sudanese PKDL [179]. Clinical trials, in Sudan, India, and Bangladesh, using the combination of AmBisome with miltefosine, have started in patients with PKDL.

Therefore, there are several challenges to be addressed for the management of PKDL. Since PKDL cases are assumed to be the carriers for transmission of VL in the Indian subcontinent, this creates a possibility for reemergence of the disease. In the present scenario, when we are talking about achieving VL elimination goal, it becomes essential to search and treat PKDL proactively.

The therapeutic regimen currently adopted for PKDL treatment requires long treatment durations and adherence for four to 12 months for a full recovery. This leads to frequent dropouts and incomplete treatment. Further, except for cosmetic consideration, PKDL does not cause physical limitations; thus, patients do not bother to seek medical care or default treatment. As stated above, the drug-associated side effects further limited patient compliance rates. Earlier, the risk of PKDL was related to incomplete SSG treatment of VL [180]; however, gradually it was realized that other treatment regimens (miltefosine, AmB, and paromomycin) [181–183] were the prolonged, toxic, and unsatisfactory outcome. The manifestation of PKDL was also reported in cases with no prior history of VL [184]. Miltefosine (12 weeks course) has been approved for PKDL; however, it has been associated with 15% to -20% relapse rates, thereby emphasizing the need to carefully monitor the drug dosage to prevent drug resistance [168, 185]. Prolonged treatment periods also possess lurking danger of emergence of drug resistance. Therefore, there remains an urgent need for more in-depth insight into the various clinic-epidemiological aspects of the disease.

## Challenges ahead: Diagnostics and detection PKDL

Management of PKDL possesses a major setback due to a lack of definitive test since rK39 positivity can be attributed to past VL infection. Also, PKDL can be a crucial player in the transmission of VL; therefore, understanding the disease pathogenesis and its control remains the key for disease management [186, 187]. The characteristic hypopigmentation in PKDL is usually misdiagnosed as vitiligo due to low parasite abundance, while the nodular form of the disease is generally confused with a vast number of dermatological conditions [188]. Currently, the gold standard for PKDL diagnosis is the documentation of parasites in slit smear or dermal tissue culture by microscopy. Similarly, detection of the parasite by PCR also has logistic issues. Further, the invasiveness of the procedure, requirement of an experienced clinician, and less sensitivity make it difficult in the endemic regions. Additionally, the refusal rates for skin biopsy is high, thus limiting its usage for diagnostics. Although detection of parasites in skin smear remains the most definitive diagnostic approach, low sensitivity rates ranging from 4% to 58% is another stumbling block [189, 190]. Usually, the low abundance of parasites associated with macular and maculopapular form of PKDL cases causes misdiagnosis, like leprosy. Currently, in endemic areas, PKDL patients are being diagnosed based on the clinical sign and symptoms, along with a previous kala-azar episode and positive antibody tests (e.g., rK39 RDT). However, parasitological confirmation using molecular techniques (such as PCR [191], restriction fragment length polymorphism (RFLP) [192], and nested PCR [193]) are highly sensitive and specific and are the only way out for the confirmed diagnosis, and thus are not practical at Primary Health Centre (PHC) levels. In addition, multiple post-PCR steps are time-consuming, requires technical expertise and it is available only in accomplished laboratories. qPCR has been another molecular tool that provides rapid and accurate diagnostic efficacy for parasite quantification [194] from DNA, isolated from a skin biopsy. In 2012, Verma and colleagues. used slit aspirates as a simple and minimally invasive tool for screening the PKDL cases, the broader application of which still needs further research [195]. In 2018, the use of SSS had proven more efficacious than biopsy microscopy, with a threshold limit of detection of four parasite per microliter in slit smear [196]. Recently, loop-mediated isothermal amplification (LAMP) assay to amplify DNA has shown to be rapid and highly specific in the field of diagnostics [197]. The technique remains a low-cost, highly sensitive tool with shorter reaction times and easy positive judgment step without the need for sophisticated molecular tools [198]. Considering these issues, there remains a considerable gap in the diagnosis; however, emerging diagnostic tools are thought to be of great help for better disease intervention in the coming days.

## Future prospects

With the current background information, the title of the present article shows a glimpse of several challenges, which should be properly and carefully encountered for sustained VL elimination. The following sections have been least explored to their potential, which can fill gaps for a complete understanding of the pathobiology of PKDL to obtain early VL elimination without stumbling WHO agenda.

### Immunology & PKDL

The disease onset, appearance, and self-healing properties greatly vary depending upon the intensity of immune responsiveness and the region to which the patient belongs. PKDL is an immunological disease where innate as well as adaptive immune cells undergo dysfunctions. The intermediate position of immune response between the Th1 and Th2 causes no systemic illness. The involvement of UV light (since lesions occur in sun-exposed areas), malnutrition, and coinfection could further drive the Th2 responses with elevated levels of Treg cell population and IL-10 production in the skin. This could be attributed to the disseminated immune responses between the skin and visceral organs leading to skin rash without systemic disease manifestation. Therefore, T-cells are a crucial determinant of immunoregulation during early immune responses. A recent report on PKDL has shown for impaired CD8+ T-cells with elevated levels of IL-10 [199]; therefore, the immunotherapeutic strategy aiming towards reinvigorating the T-cell potency needs further attention.

Furthermore, using monoclonal antibody-based approach targeting the immune checkpoint molecules and/or immunoregulatory cytokines can be another avenue that can open possibilities for therapeutic intervention. PKDL infection is associated with lack of IFNγ producing signals in the skin [200] and administration of exogenous recombinant cytokine can potentially alleviate disease pathogenesis. Therefore, immunomodulatory therapy aiming to relieve the immunosuppressive microenvironment and instigate protective Th1 responses can be used together with chemotherapeutic regimens during VL treatment to prevent the emergence of PKDL.

### Omics and PKDL

Recent advances in high throughput technologies, especially the omics tools, have revolutionized biomedical research. The collaboration of genomics with proteomics and metabolomics opens the avenue for better understanding of the causes of disease and its functional implications. The phenotypic plasticity of *Leishmania* has been looked upon by utilizing genomic, proteomic, and metabolomic approaches and has provided us with a plethora of information about its crucial role in determining the fate of infection [201, 202]. The omics studies in combination with bioinformatics could facilitate the intricacies of host–pathogen interaction for understanding the transmission cycle and improving the efficacies of existing treatment regimens. Furthermore, the utility of omics tools for enhancing the phenotypic screening and pharmacological features of upcoming candidate drugs will be of great help in the therapeutic intervention of PKDL. Although the omics technologies hold promises for its therapeutic implications, unfortunately, high costs hurdle its utility in drug discovery.

### Therapies and PKDL

The need for high investments for drug discovery and development limits its application in the field of neglected tropical diseases; therefore, judicious use of existing regimen remains the need of the hour. In this context, combination therapies have emerged as a viable option for preventing the emergence of drug-resistant parasites, improving drug efficacies, and potentially reducing the drug toxicities. Likewise, phytocompounds have also been another low-cost therapeutic option with minimal and/or no toxic effects. Plant extracts possess immunostimulatory potential. [203] and can be used in combination with existing regimens for dealing with PKDL. The advent nanocarriers for targeted drug delivery has revolutionized the search for novel therapeutic interventions. Several nanocarriers have pharmacologically being accepted as nontoxic and low-cost targeted drug delivery systems. However, their wider clinical acceptance for disease intervention needs close monitoring of their performance in terms of reduced treatment durations and robust reproducibility.

## Concluding remarks

Although considerable scientific advancements have been made in recent years, however, the emergence of drug-resistant VL cases and sequel of VL in the form of PKDL always possessed a substantial threat for achieving the goal of VL elimination. Until the elimination goals are met, it becomes crucial to judiciously employ the current chemotherapeutic strategies while keeping pace with the innovative immunotherapeutic approaches for sustaining the elimination goals. Furthermore, there remains an urgent need for stakeholders and policymakers to collaborate for improving the investment for NTDs. The close monitoring of patient compliance system can add to enhance the therapeutic indices of existing regimen while preventing the resurgence of drug-resistant and/or PKDL upon cure. The immunotherapeutic and nanotechnology-based approach can further boost the drug discovery and development process for achieving therapeutic benefits.
